# The AIP model as a theoretical framework for the treatment of personality disorders with EMDR therapy

**DOI:** 10.3389/fpsyt.2024.1331876

**Published:** 2024-01-17

**Authors:** Ad De Jongh, Laurian Hafkemeijer, Simon Hofman, Karin Slotema, Hellen Hornsveld

**Affiliations:** ^1^Research Department, PSYTREC, Bilthoven, Netherlands; ^2^Academic Centre for Dentistry Amsterdam (ACTA), University of Amsterdam and VU University Amsterdam, Amsterdam, Netherlands; ^3^School of Psychology, Queen’s University, Belfast, Ireland; ^4^Institute of Health and Society, University of Worcester, Worcester, United Kingdom; ^5^School of Health Sciences, Salford University, Manchester, United Kingdom; ^6^Department of Adult Psychiatry, GGZ Delfland, Delft, Netherlands; ^7^Department of Personality Disorders, Parnassia Psychiatric Institute, The Hague, Netherlands; ^8^Department of Clinical Psychology, Erasmus University Rotterdam, The Hague, Netherlands; ^9^Hornsveld Psychologen Praktijk, Utrecht, Netherlands

**Keywords:** personality disorder, Adaptive Information Processing model, EMDR therapy, childhood adverse events (ACEs), psychotrauma

## Abstract

Research has shown that the impact of traumatic events and circumstances on individuals is cumulative and potentially has a wide range of harmful consequences, including negative consequences on mental health. One such consequence is the development of a personality disorder, a persistent mental condition characterized by a pronounced pattern of difficulties in impulse control, emotional regulation, cognitive functions, self-esteem, and interpersonal relationships. A wide array of studies indicates that the personal history of individuals with a personality disorder is often marked by exposure to traumatic events or other types of adverse childhood experiences (ACEs). Because existing treatments for personality disorders are usually long and costly, it is essential to continue exploring alternative and complementary interventions. Nowadays, knowledge and clinical experience in regard to personality disorders have been gained in addressing ACEs by processing memories of these events through eye movement desensitization and reprocessing (EMDR) therapy. In this paper, we present a theoretical framework for this treatment approach, based on Shapiro’s Adaptive Information Processing (AIP) model, describe its current empirical basis, and provide guidance on how to formulate a useful case conceptualization that can serve as a basis for the treatment of personality disorders with EMDR therapy. This approach is illustrated with a case example.

## Introduction

Evidence suggests that some individuals become susceptible to developing subsequent psychopathological conditions of varying degrees throughout their lives (1). This susceptibility, often referred to as “latent vulnerability,” arises from a complex interplay between individual predisposition, early life experiences, and current stressful life circumstances (1). Specifically, the enduring impact of adverse childhood experiences (ACEs), including physical violence, sexual abuse, emotional abuse, and neglect, contributes to the emergence and shaping of latent vulnerability and psychopathological mental-health conditions (2, 3). Although not everyone who experiences such events will develop a mental disorder, exposure to dysfunctional situations and circumstances during childhood significantly increases the likelihood of developing psychopathology later in life (4). In this regard, there is a “dose-dependent effect,” indicating a relationship between the number of adverse events in childhood and the likelihood of developing psychopathology (4–7).

In addition to the dose-dependent relationship, evidence also suggests that the type of trauma and the age of the individual at the time of the adverse event influence psychopathology later in life (8). Likewise, the earlier abuse or neglect occurs in an individual’s life, the more severe the subsequent symptoms of psychopathology (8). Furthermore, certain critical periods in childhood appear to exist wherein experiencing a specific ACE leads to the development of specific symptoms later in life (8). For example, while exposure to an ACE meeting the DSM-5 A-criterion of post-traumatic stress disorder (PTSD; threatened death, serious injury or sexual violence) is likely to make a person particularly susceptible to PTSD, experiencing physical or emotional neglect around the age of five, has been found to increase the likelihood of developing dissociative symptoms, and when this occurs around the age of nine, it increases the likelihood of experiencing depressive symptoms. These observations form the basis of a theory called the “sensitive type and timing model” (8), and are not surprising, considering that the brain undergoes various developmental stages and associated periods of relative vulnerability [“vulnerable time windows”; (9, 10)]. In contrast, the absence of childhood trauma seems to have a protective effect. This effect is likely to be even more pronounced when positive relationships, particularly attachment relationships and healthy social interactions providing emotional stability are present. These factors are expected to buffer the impact of latent vulnerability and enhance resiliency and emotional capacity (5).

## Adverse childhood experiences and personality disorders

One way to operationalize latent vulnerability is by viewing it as a collection of (implicit) memories that can be activated under certain conditions and triggers. To this end, latent vulnerability can be seen as a subtle predisposition to later psychopathological conditions in that when new stressful experiences, such as negative peer relationships or a lack of social support, intersect with pre-existing vulnerability, the development of psychopathological conditions is likely to increase (1). A prominent example is the personality disorder.

Personality disorders are a group of mental health conditions characterized by enduring patterns of thinking, feeling, and behaving that deviate significantly from cultural expectations, causing distress or impairment in social, occupational, and other important areas of functioning. These patterns are inflexible and pervasive across various situations, and typically lead to problems in relationships and daily life. In the Diagnostic and Statistical Manual of Mental Disorders (DSM-5), personality disorders are organized into three clusters, each representing different core features: Cluster A (odd or eccentric behavior such as the paranoid personality disorder), cluster B (dramatic, emotional, or erratic behavior which includes the borderline personality disorder) and Cluster C (anxious, fearful and avoidant behavior, for example the avoidant personality disorder). Although there is no consensus on the exact etiology of personality disorders, they are generally considered to have a complex etiology involving genetic, environmental, and developmental factors. For example, although it is widely accepted that genetics plays a role in the development of personality disorders (11), it is important to note that the precise genetic mechanisms and their contribution to these disorders are still being investigated. Furthermore, it should be noted that not all individuals with personality disorders report a history of trauma and not all individuals with ACEs develop personality disorders (12).

It is well established that child maltreatment is prevalent and strongly associated with nearly all types of personality disorders (13, 14). Up to 85% of individuals with a personality disorder report some form of negative childhood experience (15), including both abuse (73%) and neglect [82%; (16)]. The relationship between childhood experiences and personality disorders varies depending on the specific personality disorder (17). For instance, borderline personality disorder has been found to be most strongly associated with ACEs. Research indicates that individuals with this mental health condition report an average of 13 times more ACEs than individuals without this disorder (18).

One mechanism potentially contributing to the manifestation of personality disorders, is that ACEs can have profound and lasting effects on neurobiological development. Exposure to childhood adversities and chronic stress have been linked to alterations in the structure and function of the hippocampus, amygdala, and prefrontal cortex. These areas are crucial for memory formation, emotional regulation, decision-making, and social behavior. Results of a recent meta-analysis clearly show that a history of adverse childhood events is associated with long-lasting increases in amygdala responses in adults and reduced prefrontal cortex responses (19). Changes in these structures are likely to contribute to difficulties in processing and regulating emotions seen in some personality disorders.

## Adaptive Information Processing (AIP) model

Findings from scientific research on latent vulnerability [e.g., (18)] can be closely linked to the AIP model, which serves as the foundation for EMDR therapy (20, 21). This theory posits that many forms of psychopathology, with PTSD as the most notable example, stem from disruptive life experiences. According to the AIP model, these experiences are consolidated into neural networks as distressing mental images, dysfunctional cognitions, negative emotions, and physical sensations. Furthermore, Shapiro’s AIP model suggests that the intense affect associated with a traumatic event disrupts normal information processing: ‘Target events remained unprocessed because the immediate biochemical responses to the trauma have left it isolated in neurobiological stasis’ [(20), p. 338]. The AIP model predicts that adverse events throughout life are stored as unprocessed memories, which can then be triggered and activated by specific environmental circumstances. In fact, this dynamic is conceptually similar to the concept of latent vulnerability.

Another premise of the AIP model is that each individual possesses an innate information-processing system that enables adaptive learning from new experiences. Through the use of EMDR therapy, it is believed that dysfunctional stored information can be activated in a way that enables connections with existing networks of functional information and healthy beliefs (21). This process is thought to transform traumatic memories into a more adaptive and functional form, leading to a change in negative personal meaning regarding the traumatic experience and subsequent symptom reduction (21).

## The dynamics of personality disorders in light of the AIP model

**Figure 1:**
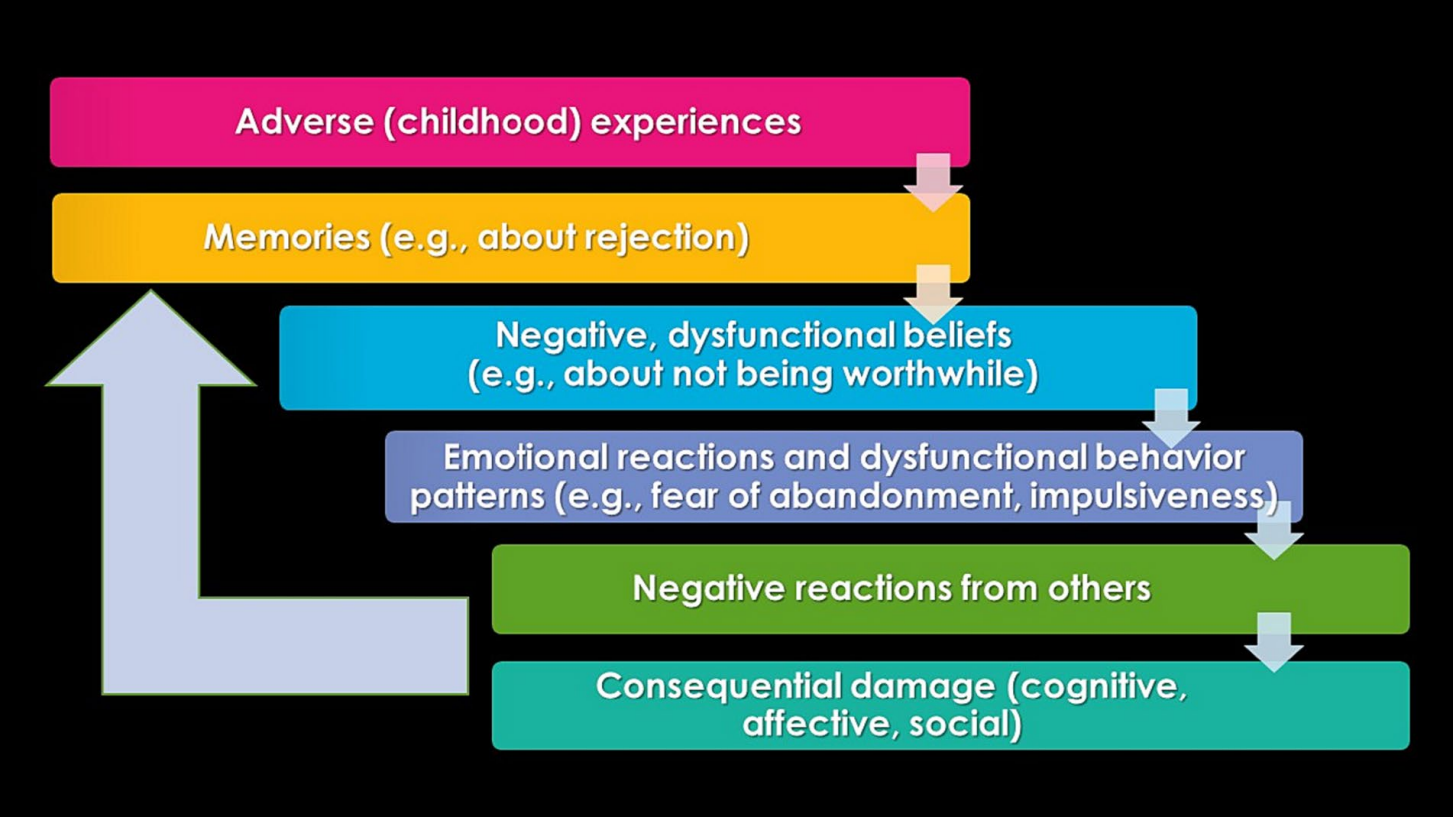
A schematic representation of the adapted AIP model described here illustrates a self-reinforcing pattern of latent vulnerability in relation to the development of personality pathology.

Figure 1 provides a more detailed and expanded representation of the AIP model, drawing from scientific knowledge and experiences with EMDR therapy in clinical practice for personality disorders (22–24). It illustrates the reactivation of specific parts of an individual’s neural network that manifest as trauma-related symptoms, including negative self-perceptions and strong uncontrollable emotional reactions. This can be expressed as fear of abandonment, mistrust, or other forms of dysfunctional interpersonal behavior, leading to conflicts with the individual’s environment and important others thereby contributing to a (further) development of personality pathology. Figure 1 also shows that interpersonal conflicts may give rise to new negative memories and a self-reinforcing cycle of negative beliefs, emotional reactions, and dysfunctional behavioral patterns, with potential adverse consequences for individuals at the cognitive, affective, and social levels. An unfortunate consequence is that the likelihood of further damage in the form of avoidance of social interactions and social isolation (“social thinning”) increases (25).

## The relationship between the classifications of PTSD, complex PTSD, and personality disorders

PTSD is relatively common in personality disorders (26). For example, in individuals with DSM-IV cluster C personality disorder, more than one-third of them appear to fulfill the diagnostic criteria for PTSD (27). However, the relationship between trauma, PTSD, and borderline personality disorders is particularly striking. Borderline personality disorder is characterized by a profound pattern of instability in interpersonal relationships, low self-esteem, and emotion regulation problems, beginning in adolescence and manifesting in various situations [DSM-5, (28)]. While 30–70% of adults diagnosed with borderline personality disorder also meet the criteria for PTSD at some point in their lives, conversely, 25–30% of adults with PTSD may also be diagnosed with borderline personality disorder (29).

The introduction of the relatively new classification of Complex PTSD and revised descriptions of personality disorders in the ICD-11 certainly have not simplified the making of a (differential) diagnosis. This is mainly due to the similarities between the diagnostic profiles of Complex PTSD and borderline personality disorder, which is identified as a personality disorder with the specification “borderline pattern” in the ICD-11 (30). Particularly in the areas of emotion regulation, interpersonal relationships, and the presence of negative self-perceptions, negative perceptions of others, or negative perceptions of the world, this created significant overlaps between the symptom clusters of both conditions (30, 31).

## The treatment of personality disorders

Given that some of the characteristic symptoms of personality disorders overlap with those of (Complex) PTSD, it is noteworthy that treatment guidelines for these disorders differ significantly. For the treatment of PTSD, a trauma-focused treatment approach is recommended, consisting of 8–12 sessions of trauma-focused cognitive behavioral therapy or EMDR therapy (32). In contrast, psychotherapy is the preferred treatment for borderline personality disorder, with an explicit recommendation against short psychological interventions lasting less than 3 months (33).

Currently, various therapies for personality disorders have been studied and recognized as effective, with no treatment method proving superior (34). Most of these therapies focus on addressing problems characteristic of personality disorders, such as the transfer of unconscious feelings and conflicts within a therapeutic relationship [Psychodynamic psychotherapy; (35)], promoting mentalization and learning to regulate emotions [Mentalization-based Therapy; (36)], restructuring deep-rooted dysfunctional schemas and behavioral patterns [Schema-focused therapy; (37)], or reducing self-destructive behavior and promoting emotion regulation [dialectical behavior therapy; (38)]. However, a therapy that focuses purely primarily on processing pathogenic memories of ACEs that are believed to be responsible for the development and maintenance of the personality disorder is not yet generally promoted.

## Empirical support for the effect of trauma-focused treatment in individuals with borderline personality disorder

The field of personality disorders has long been hesitant regarding trauma-focused treatment approaches in individuals with both borderline personality disorder and PTSD resulting from multiple traumas (39). This is primarily due to the limited emotion regulation skills and increased suicide risk characteristic of this population. While understandable, such recommendations can result in not receiving or inadequately receiving trauma-focused treatment (40). However, various studies reveal that trauma-focused treatment for PTSD in individuals with a (borderline) personality disorder is feasible and safe (41–43). These findings are further in line with those of a meta-analysis of 12 studies involving patients predominantly diagnosed with borderline personality disorder and PTSD that showed significant reductions in both PTSD symptoms and general psychopathological symptoms (44). Moreover, no increase in negative side effects, such as suicide attempts, severe self-harming behavior, or hospitalizations, have been reported. In addition, the dropout rate was relatively low (17%). The authors therefore assert that “Psychotherapy for PTSD is efficacious and safe for patients with borderline personality disorder and should not be withheld from these vulnerable individuals” [(44), p. 1].

The information presented so far primarily pertained to research on the effects associated with the treatment of PTSD in individuals with borderline personality disorder. To our knowledge, only six studies have been published to date that have investigated the treatment of PTSD and its effect on the symptoms of borderline personality disorder itself (45). One controlled efficacy study focused on the treatment of individuals within an integrated dialectical behavioral therapy-PTSD-borderline treatment program (46), and two controlled studies (47–49) on the effectiveness of Narrative Exposure Therapy (NET). These four studies found a significant reduction in symptoms characteristic of borderline personality disorder. Two other uncontrolled studies examined the effects of intensive trauma-focused treatment for PTSD on borderline symptoms (50, 51). Both studies involved an eight-day treatment consisting of eight sessions of imaginal exposure and *in vivo* exposure lasting 90 min and eight 90-min sessions of EMDR therapy, supplemented with psychoeducation and physical activities. One of these studies, with a sample of 45 patients diagnosed with both PTSD and borderline personality disorder, found that 1 year after treatment, 73% of the patients no longer met the diagnostic criteria for borderline personality disorder (51).

It is noteworthy that to date, only one study has been conducted in which a trauma-focused treatment was applied to individuals with a personality disorder *without* comorbid PTSD (24). This study, conducted within an outpatient mental health care institution involving 97 patients with a personality disorder, explicitly excluded those with comorbid PTSD. The treatment group received five weekly 90-min sessions of EMDR therapy, while the control group consisted of individuals on a 5-week waiting list. Both groups subsequently received treatment as usual for personality disorders. General functioning and personality dysfunction decreased significantly and more rapidly in the EMDR group than that in the control group. These results were still maintained 3 months after the start of the treatment. The dropout rate was remarkably low (9%), and the treatment duration (five weekly EMDR therapy sessions) was notably short, much shorter than in other therapies for personality disorders [e.g., dialectical behavior therapy; (52)]. In summary, the results of this study suggest that EMDR therapy is both an effective and efficient therapeutic modality and can play a significant role in the treatment of individuals with personality disorders, even in the absence of comorbid PTSD.

## Case conceptualization

EMDR therapy focuses on processing pathogenic memories or other mental representations such as fantasized images (53), which have contributed to the development and maintenance of the disorder (Level 2 in Figure 1). The central assumption is that targeting these memories leads to a significant reduction in symptoms and, thus, maximizes the patient’s quality of life (21). In fact, if it holds true that experiencing more ACEs leads to increased pathology, the reverse might also be true. This means that the more pathogenic memories can be reprocessed, the fewer symptoms will remain, and the better an individual will function. To select the memories crucial for influencing the symptom clusters of personality disorders, thorough trauma-sensitive case conceptualization is a fundamental starting point. This aspect of therapy aims to describe a plausible connection between existing symptoms and meaningful memories that are believed to drive the pathology, and to create a treatment plan based on addressing the selected memories.

Based on experiences from the first randomized controlled trial of trauma-focused treatment for personality disorders (24) and the experience of (intensive) treatment of Complex PTSD with or without comorbid personality pathology (23, 50, 51, 54, 55), we developed a step-by-step plan that can help identify, organize, and desensitize crucial memories using trauma-focused therapies, including EMDR therapy. In EMDR therapy, clients are guided through the desensitization of a memory, typically by applying lateral hand movements. EMDR 2.0, a novel version of EMDR therapy, that capitalizes on the scientific research into working memory theory (56) uses a variety of additional tasks to maximally tax clients’ working memory, like complex eye movements or the spelling of words. While a study found no overall superiority, EMDR 2.0 showed efficiency, requiring fewer sets for comparable reductions in emotionality and vividness of traumatic memories (57).

The model used for the treatment of personality disorders consists of six steps. First, intrusive memories that meet the A-criterion of the DSM-5 classification for PTSD are identified (Step 1). Subsequently, other pathogenic memories related to A-criterion events from the DSM-5 are selected, as well as intrusive memories that do not meet the A-criterion (Step 2). In Step 3 patient’s most prominent symptom clusters are identified while in Step 4, the memories that gave rise to or believed to have perpetuated these primary complaints are addressed. In Step 5 the sequence for the desensitization of the memories is determined, and in Step 6 the Standard EMDR protocol is carried out (Tables 1, 2).

**Table 1:** The six-step treatment model for the treatment of personality disorders.

1. Identify the patient’s intrusive memories that meet the A-criterion of the DSM-5 classification for PTSD.^1^
2. Identify both non-intrusive memories of A-criterion-worthy events and intrusive memories of non-A-criterion-worthy events.
3. Identify the patient’s most prominent symptom clusters, such as lack of self-esteem. Emotional regulation problems, interpersonal mistrust, and fear of abandonment.
4. Identify the memories that gave rise to or significantly exacerbated these symptoms. For this purpose it is important to conduct a specific search during early childhood. The use of the prompts in Table 2 can be helpful. Organize all memories based on the age at which the event occurred, placing them on an imaginary timeline.
5. Determine the sequence for desensitizing memories. Begin with memories identified in Steps 1 and 2. Create a list of memories, with the order determined by the Subjective Units of Disturbance (SUD) of the (whole) memory. The higher the SUD score, the higher the memory is placed on the list of memories to desensitize. The list is supplemented with the memories identified in Step 4. The goal of Step 4 was to firstly address the symptom cluster that causes the most distress to the patient, whereas in Step 5 memories are organized based on the SUD score for the entire memory. Memories with equal SUDs are arranged by age, with memories of events at a younger age addressed first.
6. Treat the memories using the EMDR Standard Protocol, starting with the memory at the top of the list.

^1^This step should not belong to the treatment of a personality disorder, but precede it. However, we included this step in our model because PTSD and partial PTSD referred to for personality treatment are often unrecognized.

**Table 2:** Useful prompts for identifying crucial mental representations for therapy.

‘When or what caused these problems to start, and when or what made them worse?’
‘Which situations from your early childhood still emotionally prove to you that you are not worthwhile/cannot trust people?’
‘Which memory from your early childhood do you need to evoke so that you now feel again maximally angry/guilty/ashamed?’
In case of anticipatory anxiety, specifically fear of rejection, the client’s flashforward can be targeted: “What is the most terrible thing that could happen in your fantasy?.”

## A case description

Ayla is a 29-year-old woman who participated in a randomized controlled trial (RCT) on the effectiveness of EMDR treatment in individuals with personality disorder [TEMPO study; (58)]. She presented to the emergency department of a mental health institution because of depressive symptoms and fatigue. Ayla indicated that she grew up in an environment characterized by emotional neglect, both physical and verbal abuse, and unwanted sexual experiences. After turning 21, she was physically abused and threatened with death by her then-partner. Due to harassment and violent incidents, she received treatment in various mental health institutions from the age of 16–21 years old, with over 75 treatment sessions, primarily consisting of supportive and insight-oriented psychotherapy, as well as family sessions and emotion regulation training. She had not previously received any trauma-focused treatment. During the intake assessment, Ayla met the diagnostic criteria for PTSD (CAPS-5 score 49), and the structured clinical interview for DSM-5 personality disorders (SCID-5-P) indicated that she particularly struggled with emotion regulation problems and fear of abandonment, and fulfilled the DSM-5 criteria for borderline personality disorder. During the first session, a case conceptualization was established using the structure mentioned above. Ayla reported one A-criterion event (#1 physical violence with death threat by partner) as being intrusive and five other A-criterion events that were not intrusive. She indicated her emotion regulation problems as the most prominent symptom cluster and reported 11 memories as the cause. With fear of abandonment being the second most prominent complaint that bothered her, Ayla, with the help of the therapist, was able to find two memories that had caused or worsened these complaints. Thus, a total of 19 memories required processing.

**Figure 2:**
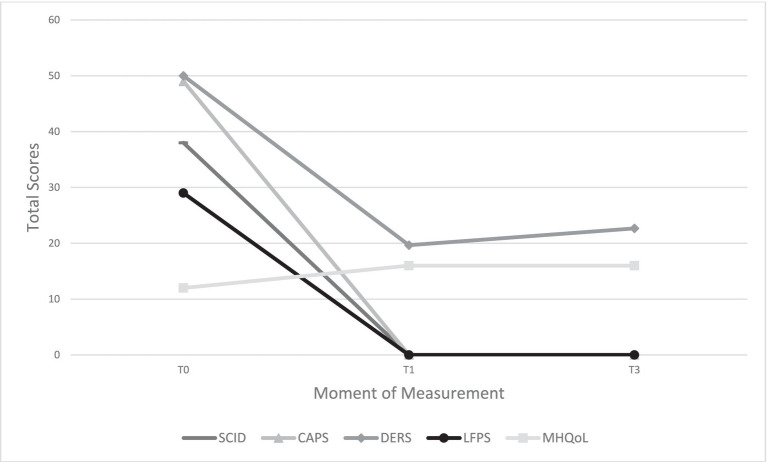
Different outcome measures at three measurement moments. SCID, Structured Clinical Interview for DSM-5 Personality Disorders; CAPS, Clinician-Administered PTSD scale for DSM-5; DERS, Difficulties in Emotion Regulation Scale; LFPS, Level of Personality Functioning Scale; MHQoL, Mental Health Quality of Life; T0, before treatment; T1, after treatment; T3, three months after treatment.

As EMDR targeting memory #1 began, the SUD score decreased from 10 to 7 but then increased again during “back to target.” By adding additional working memory load, buzzers, naming colors, and spelling tasks [according to the approach of EMDR 2.0; (57)], the memory was desensitized (SUD = 0). The second memory (pregnancy toxemia) could also be desensitized. At the end of the session, Ayla reported feeling relieved, less tension, and no physical pain. At the beginning of the second EMDR therapy session, Ayla felt cheerful and relaxed and expressed confidence in the rest of the treatment. While EMDR therapy progressed smoothly in the following five sessions, the SUD-score sometimes decreased slowly. This was most likely because Ayla often blamed herself for events and drew negative conclusions about herself as a person. The use of cognitive interweaves (e.g., “Do you really think a 7-year-old girl could have stopped the violence?”) helped to break this pattern, after which Ayla began to see herself differently. She began to doubt the messages she received from her parents as a young child and gradually started to view herself as a strong woman. In subsequent sessions, all memories were processed. In the seventh session, any remaining symptoms were assessed and the memories driving these symptoms were identified. Ayla mentioned still struggling with a fear of rejection, and four new memories related to false accusations and conflicts with loved ones could be identified. These memories were then processed during the same session. At the end of the seventh session, Ayla reported no complaints, and expressed confidence in facing previously avoided situations. After treatment and 3 months later, Ayla no longer met the diagnostic criteria for borderline personality disorder according to the SCID-5-P and PTSD (CAPS score = 0). Figure 2 depicts the course of personality disorder symptoms, PTSD symptoms, the course of emotion regulation problems, and the level of quality before treatment, immediately after treatment, and 3 months after EMDR therapy.

## Further treatment

Breaking down the coping and survival strategies typically present in patients with personality disorders, as outlined in step 4 of Figure 1, can be a challenging task. Therefore, it is crucial for the therapist to take time at the end of each session to focus on the theme of the symptom cluster and discuss the associated patterns. The therapist can facilitate this process by asking questions such as “What does what you have learned in this session mean for your daily life? How can you approach things differently?” The more concrete the patient understands how to implement these changes, the greater the expected effect. This can be done, for example, by asking, “Could you please outline the scenario and how do you envision it?” If obstacles emerge during this conversation that perpetuate avoidance behaviors, such as fears of abandonment or rejection, it is wise to address them immediately. Within EMDR therapy, this can be achieved by using EMDR targeting catastrophic scenarios (“flashforwards”), conducting mental video checks, future templates, and behavioral experiments to break avoidance patterns (59).

## Discussion and conclusion

In recent years, research has shown that traumatic events and circumstances have a cumulative impact on individuals, potentially leading to a wide range of harmful consequences, including mental health issues (7). One of these is the development of a personality disorder, a mental health condition that often causes high levels of distress and significantly impairs a person’s quality of life. The existing treatments for personality disorders are usually long and costly. Therefore, it is essential to continue exploring alternative interventions that are ideally shorter in duration. A trauma-focused approach using EMDR therapy, as well as other therapies aimed at trauma processing (e.g., imagery rescripting), could offer this. Clearly, the choice of a specific therapy will depend on various factors, including the nature and severity of personality pathology, individual needs and abilities, and available therapeutic expertise.

In this paper, we have attempted to provide a novel framework for the application of EMDR therapy in people with a personality disorder. This is relevant given the strong empirical foundation for this therapy as a first-line treatment for the processing of disturbing memories of adverse events (60, 61). The currently limited available literature on the effectiveness of trauma-focused psychotherapy supports the notion that EMDR therapy is a feasible, safe, and effective treatment option for personality disorders (24, 51).

As a treatment philosophy and associated therapeutic framework for personality pathology, Shapiro’s AIP model has proven to be a valid foundation. The AIP model posits that negative self-beliefs (e.g., “I am not good enough”), emotion regulation problems, interpersonal problems or other core features of personality disorders are not seen as the cause of present dysfunction, but symptoms of unprocessed, inadequately stored memories of earlier life experiences that contain that affect and perspective. From this perspective, a personality disorder can be considered a collection of symptoms, the origins of which can be traced back to adverse childhood events on a lifeline. Although it is primarily a descriptive model that lacked empirical support when it was introduced (62), the framework in which we were able to apply EMDR therapy today to treat personality pathology (23, 24, 51), is a clear example of a substantive domain that is suitable for further elaboration and empirical support of the AIP model. Furthermore, the concept of “latent vulnerability” proves not only useful and insightful for therapists, but can also be used as a basis for psychoeducation. In addition, the connection to research on the ACEs provides a developmental psychological perspective, strengthens the foundation of the AIP model, and shifts it from a purely theoretical framework to a testable one. To this end, the results of a controlled outcome study, investigating the long-term effects of the treatment model described in this paper will not only determine whether the vision on personality pathology described here is sustainable and valid (58), but also whether in the future, EMDR therapy may become a guideline treatment for both PTSD and personality disorders.

## Author contributions

AJ: Conceptualization, Investigation, Project administration, Writing – original draft, Writing – review & editing. LH: Investigation, Writing – review & editing. SH: Writing – review & editing. KS: Writing – review & editing. HH: Writing – review & editing.
